# Bayesian Analysis of Predictors of Incomplete Vaccination against Polio among Children Aged 12–23 Months in Ethiopia

**DOI:** 10.3390/ijerph182211820

**Published:** 2021-11-11

**Authors:** Teshita Uke Chikako, Abdul-Aziz Seidu, John Elvis Hagan, Richard Gyan Aboagye, Bright Opoku Ahinkorah

**Affiliations:** 1 Wondo Genet College of Forestry and Natural Resource, Hawassa University, Hawassa P.O. Box 05, Ethiopia; teshitauke@hu.edu.et; 2 College of Public Health, Medical and Veterinary Sciences, James Cook University, Townsville, QLD 4811, Australia; abdul-aziz.seidu@stu.ucc.edu.gh; 3 Department of Estate Management, Takoradi Technical University, Takoradi P.O. Box 256, Ghana; 4 Centre For Gender and Advocacy, Takoradi Technical University, Takoradi P.O. Box 256, Ghana; 5 Department of Health, Physical Education and Recreation, University of Cape Coast, Cape Coast TF0494, Ghana; 6 Neurocognition and Action-Biomechanics-Research Group, Faculty of Psychology and Sport Sciences, Bielefeld University, Postfach 100131, 33501 Bielefeld, Germany; 7 Department of Family and Community Health, School of Public Health, University of Health and Allied Sciences, Ho PMB 31, Ghana; raboagye18@sph.uhas.edu.gh; 8 School of Public Health, Faculty of Health, University of Technology Sydney, Sydney, NSW 2007, Australia; bright.o.ahinkorah@student.uts.edu.au

**Keywords:** Bayesian logistic regression analysis, childhood vaccination, Ethiopia, polio, predictors

## Abstract

Background: The re-introduction of polio among children aged 12–23 months is likely to occur in Ethiopia due to the low vaccination rates against poliovirus. The study sought to examine the predictors of incomplete vaccination against polio among children aged 12–23 months in Ethiopia. Methods: The data used were obtained from the 2016 Ethiopia Demographic and Health Survey. Binary and Bayesian logistic regressions were used for the data analysis, with parameters estimated using classical maximum likelihood and the Bayesian estimation method. Results: The results revealed that 43.7% of the children were not fully vaccinated against polio in Ethiopia. Maternal age, educational level, household wealth index, exposure to mass media, place of residence, presence of nearby healthy facility, counseling on vaccination, and place of delivery were significant determinants of incomplete polio vaccination among children aged between 12 and 23 months in Ethiopia. Conclusion: Considerable numbers of children are not fully vaccinated against polio in Ethiopia. Individual and contextual factors significantly contributed to incomplete polio vaccination among children in the country. Therefore, the government and other stakeholders should pay particular attention to maternal education to increase mothers’ educational level in all regions and give training and counseling in all urban and rural parts of the country on child vaccination to overcome the problem of children’s incomplete polio vaccination and/or vaccination dropout.

## 1. Introduction

Vaccination is a simple, safe, and effective way of protecting children against infectious diseases before they come into contact with them. Immunization is a worldwide health and improvement success history, saving millions of children’s lives every year and reducing the risk of getting a disease by working with your body’s natural defenses to build protection [1]. Immunization is the constituent of primary health care and an indubitable human right and it is critical to the prevention and control of infectious disease outbreaks. They also underpin global health security and will be a vital tool in the battle against antimicrobial resistance [2].

Globally, about two to three million childhood deaths annually could be averted through the use of vaccines [3,4]. Each year, approximately twenty million children fail to take all recommended vaccines, and almost two-thirds of these children come from just ten countries, including Ethiopia [4,5]. According to the World Health Organization’s (WHO) guidelines, children are considered as fully vaccinated against polio when they have received all recommended vaccination doses by the age of 12–23 months [6,7,8]. Poliovirus cases had reduced by over 99% since 1988, from a projected 350,000 cases in more than 125 endemic countries to 179 described cases in two countries in 2019 [9]. As a result of global eradication efforts, polio is now only endemic in Afghanistan, Nigeria, and Pakistan [10,11]. However, countries with low vaccination rates continue to be at risk for re-introduction of the virus due to imported cases resulting from travel. Unvaccinated travelers, especially children, pregnant women, and those with weakened immune system going to areas with polio outbreaks are at risk [11]. However, countries that have been cleared of the wild poliovirus, but at low vaccination rates and with weak and porous surveillance systems, continue to be at risk for re-introduction of the virus due to imported cases [12]. Again, surveillance evolves according to epidemiological changes and the indications on the performance of the surveillance system given by WHO may be susceptible to the integration of further systems of analysis [13]. 

The Ethiopia Demographic and Health Survey (EDHS) indicates that the percentage of children aged 12–23 months who are fully vaccinated increased by 15%, from 24% in 2011 to 39% in 2016 [14]. The Expanded Programme on Immunization (EPI) was established by the WHO in 1974 to control vaccine-preventable diseases [15]. Ethiopia has been realizing EPI free of charge aiming to immunize all children between the ages of 0 and 23 months against oral polio [16]. The findings of a recent study on factors associated with non-vaccination against polio has awakened the need for research to understand the vaccination status of children against polio in specific countries in Africa [11]. An extensive search of the literature on childhood vaccination in Ethiopia, where polio constitutes a major cause of death among children under five, shows that most studies conducted on childhood vaccination focused on the basic childhood vaccines together to determine whether a child was fully vaccinated or not. This approach makes it difficult to understand what the situation is with specific vaccines such as the polio vaccines. Due to inadequate information on childhood vaccination against polio in Ethiopia, the severity and the descriptions on it are quite scanty. Therefore, the purpose of the paper was to examine the factors associated with incomplete vaccination against polio among children aged between 12 and 23 months in Ethiopia to make recommendations that will help eradicate poliovirus from the country. 

## 2. Materials and Methods

### 2.1. Source of Data

The data used for this study were obtained from the 2016 version of the EDHS on the polio vaccination status of children aged between 12 and 23 months. The sampling frame used for the 2016 EDHS is the Ethiopia Population and Housing Census (PHC), which was conducted in 2007 by the Ethiopia Central Statistical Agency. Administratively, Ethiopia is divided into nine geographical regions and two administrative cities. The sample for the 2016 EDHS was designed to provide estimates of key indicators for the country as a whole, for urban and rural areas separately, and for each of the nine regions and the two administrative cities. The survey was conducted from 18 January 2016 to 27 June 2016 [13]. Details of the EDHS methodology and sampling have been published in the EDHS report, which is also available online at https://dhsprogram.com/publications/publication-fr328-dhs-finalreports.cfm?cssearch=351226_1 (accessed on 25 July 2021).

### 2.2. Variables of the Study

#### 2.2.1. Outcome Variable

Polio vaccination status was the outcome variable. Considering the WHO protocol, incomplete vaccination can be defined as children who missed at least one dose of the four vaccines between 12 and 23 months [5,6]. Polio vaccination was captured as a dichotomous variable and coded as 1 if a child aged between 12 and 23 months has missed at least one dose of the four basic doses of oral polio vaccine (polio 0 at birth, polio 1 at 6 weeks, polio 2 at 10 weeks, and polio 3 at 14 weeks) and 0 if otherwise [7,17,18,19]. 

#### 2.2.2. Explanatory Variables

The explanatory variables used for this study were sex of the child (male and female), maternal age (15–24, 25–34, and 35–49), wealth index (poor, middle, and rich), maternal occupation (housewife, employee, daily labor market, and merchant) and educational level (no education, primary, and secondary/above). Other variables were exposure to mass media (not at all, once a week, and twice and more than a week), family size (1–3, 4–6, and 7 and above), place of residence (urban and rural), presence of nearby health facility (yes and no), type of nearby health facility (health center, health post, and private clinic), means of transport (on foot and transportations), counseling on vaccination (yes and no), and place of delivery (health facility and home). These variables were considered based on previous studies which found them associated with vaccination status among children aged between 12 and 23 months [7,17,18,19].

### 2.3. Data Analyses

#### 2.3.1. Binary Logistic Regression

Binary logistic regression allows one to predict a dichotomous outcome, from a set of predictor variables that may be continuous, discrete, dichotomous, or a mix of any of these [20,21,22]. Therefore, this study used binary logistic regression; because the response variable, polio vaccination status has two categories or is dichotomous. The response variable was given as follows

$$y_{i}=\{\begin{array}{c} 1=if\;the\;i^{th}\;child\;was\;polio\;fully\;vaccinated\\ 0=if\;the\;i^{th}\;child\;was\;not\;fully\;vaccinated \end{array}.$$

Then, the conditional probability that the respondent’s polio vaccinated status given the X set of predictor variables is denoted by Prob ($Y_{i}$ = 1 ∣ X) = $P_{i}$. The expression $P_{i}$ has the form:

$$P_{i}=\frac{e^{\beta_{0}+\beta_{1}X_{1i}+\beta_{2}X_{2i}+\beta_{3}X_{3i}+\cdots +\beta_{k}X_{ki}}}{1+e^{\beta_{0}+\beta_{1}X_{1i}+\beta_{2}X_{2i}+\beta_{3}X_{3i}+\cdots +\beta_{k}X_{ki}}}=\frac{e^{X^{\prime }\beta }}{1+e^{X^{\prime }\beta }}$$

where $P_{i}$ is the probability of child *i* is not fully vaccinated, *Y_i_* is the observed polio fully non-vaccination status of a child, and β is a vector of fixed unknown coefficients.

The parameter was estimated by using the maximum likelihood estimation method. The estimated coefficients of variables, $\hat{\beta }$, the standard errors of estimates S.E($\hat{\beta }$), Wald statistic, significance, the odds ratio (Exp($\hat{\beta }$)), and the 95% confidence interval for all predictor variables in the model are presented in the results section. The standard error of the estimate helps in computing the values of the Wald statistic, the significance of the Wald statistic indicates the importance of the predictor variable in the model, and the high value of the Wald statistic shows that the corresponding predictor variable was important. Since the covariates used in the model are categorical, to compute the odds ratio, a reference category (ref) was needed. The exp ($\hat{\beta }$) is called the odds multiplier or odds ratio with an odds ratio of 1 for the reference category, and all other groups are compared based on the reference group.

#### 2.3.2. Bayesian Logistic Regression

In Bayesian analysis, the three key ingredients are the likelihood function, which reflects information about the parameters contained in the data, the prior distribution, which quantifies what is known about the parameters before observing data, and the prior distribution and likelihood can be easily combined to form the posterior distribution, which represents total knowledge about the parameters after the data have been observed [23,24,25].

#### 2.3.3. The Likelihood Function

In this study, Bayesian logistic regression specifies a dichotomous dependent variable as a function of a set of explanatory variables. The likelihood input from the $i^{th}$ subject follows Bernoulli distribution and given by:

$$Bernoulli\;\left(P_{i}\right)=P_{i}^{y_{i}}{\left(1-P_{i}\right)}^{1-y_{i}},\;\beta_{i}\sim N\left(\mu_{i}\;,\sigma_{i}^{2}\right)$$

where $\pi_{i}$ represents the probability of the event for subjects *i* who have covariate vector $x_{i}$, and $y_{i}$ indicates the presence, $y_{i}$ = 1, or absence $y_{i}$ = 0 of the event for that subject.

$$\mathrm{In}\;\mathrm{logistic}\;\mathrm{regression},\;\mathrm{it}\;\mathrm{is}\;\mathrm{known}\;\mathrm{that}:\;P_{i}=\frac{e^{\beta_{0}+\beta_{1}X_{1i}+\beta_{2}X_{2i}+\cdots +\beta_{k}X_{ki}}}{1+e^{\beta_{0}+\beta_{1}X_{1i}+\beta_{2}X_{2i}+\cdots +\beta_{k}X_{ki}}}$$

where $P_{i}$ = the probability of $i^{th}$ child not fully vaccinated against polio, so that the likelihood contribution from the $i^{th}$ subject is given by

$$L\left(y│\beta \right)={\left(\frac{e^{\beta_{0}+\beta_{1}X_{1i}+\beta_{2}X_{2i}+\cdots +\beta_{k}X_{ki}}}{1+e^{\beta_{0}+\beta_{1}X_{1i}+\beta_{2}X_{2i}+\cdots +\beta_{k}X_{ki}}}\right)}^{y_{i}}{\left(1-\frac{e^{\beta_{0}+\beta_{1}X_{1i}+\beta_{2}X_{2i}+\cdots +\beta_{k}X_{ki}}}{1+e^{\beta_{0}+\beta_{1}X_{1i}+\beta_{2}X_{2i}+\cdots +\beta_{k}X_{ki}}}\right)}^{1-y_{i}}.$$

Since individual children are assumed to be independent, then, the likelihood function over a data set of n subjects is given by

$$L\left(y│\beta \right)=\prod_{i=1}^{n}\left[{\left(\frac{e^{\beta_{0}+\beta_{1}X_{1i}+\beta_{2}X_{2i}+\cdots +\beta_{k}X_{ki}}}{1+e^{\beta_{0}+\beta_{1}X_{1i}+\beta_{2}X_{2i}+\cdots +\beta_{k}X_{ki}}}\right)}^{y_{i}}{\left(1-\frac{e^{\beta_{0}+\beta_{1}X_{1i}+\beta_{2}X_{2i}+\cdots +\beta_{k}X_{ki}}}{1+e^{\beta_{0}+\beta_{1}X_{1i}+\beta_{2}X_{2i}+\cdots +\beta_{k}X_{ki}}}\right)}^{1-y_{i}}\right].$$

#### 2.3.4. The Prior Distribution

The prior distribution was a probability distribution that indicates the previous information related to the parameters of concern. In this study, a non-informative prior normal distribution was used, and the assigned normal prior distribution for the components of $\beta ={\left(\beta_{0},\beta_{1},\;\cdots ,\;\beta_{k}\right)}^{\prime }\;was:$

$$f\left(\beta_{j}\right)=\overset{k}{\underset{j=0}{\prod }}\frac{1}{\sqrt{2\pi \delta_{j}^{2}}}exp\left\{\frac{-1}{2}{\left[\frac{\beta_{j}-\mu_{j}}{\delta_{j}}\right]}^{2}\right\}$$

where the common choice for µ is zero, and δ is usually chosen to be large enough to be considered as non-informative, common choices being in the range from δ = 10 to δ = 100.

#### 2.3.5. The Posterior Distribution

From a specified likelihood of logistic regression and the prior distribution above, the posterior distribution of the Bayesian logistic regression contains a combination of both types of information, which were obtained from data (likelihood) and previous information obtained from the study area before data were collected (prior). Therefore, the posterior distribution was derived by multiplying the prior distribution over all parameters by the full likelihood function. Then, the posterior distribution was written in the form of:

$$\begin{array}{c} f\left(y|\beta \right)=L\left(y|\beta \right)*f\left(\beta \right)=\\ \prod_{i=1}^{n}\left[{\left(\frac{e^{\beta_{0}+\beta_{1}X_{1i}+\beta_{2}X_{2i}+\cdots +\beta_{k}X_{ki}}}{1+e^{\beta_{0}+\beta_{1}X_{1i}+\beta_{2}X_{2i}+\cdots +\beta_{k}X_{ki}}}\right)}^{y_{i}}{\left(1-\frac{e^{\beta_{0}+\beta_{1}X_{1i}+\beta_{2}X_{2i}+\cdots +\beta_{k}X_{ki}}}{1+e^{\beta_{0}+\beta_{1}X_{1i}+\beta_{2}X_{2i}+\cdots +\beta_{k}X_{ki}}}\right)}^{1-y_{i}}\right]*\prod_{j=0}^{k}\frac{1}{\sqrt{2\pi \delta_{j}^{2}}}exp\left\{\frac{-1}{2}{\left[\frac{\beta_{j}-\mu_{j}}{\delta_{j}}\right]}^{2}\right\} \end{array}$$

Bayesian logistics model inference was based on samples drawn from the posterior distribution using the Gibbs sampler algorithms. The Gibbs sampling algorithm is one of the simplest Markov chain Monte Carlo (MCMC) algorithms that converge to the target density as the number of iterations become large. The posterior means of the parameters was estimated based on 50,000 iteration of MCMC samples.

### 2.4. Software Used

The statistical software used in this study was SPSS version 20 and WinBUGS14. SPSS was used for the descriptive and binary logistic analysis, and WinBUGS14 was used for the Bayesian analysis. The Bayesian logistic regression analysis using the Gibbs sampling method in the WinBUGS software was considered. The Bayesian model used normal–normal, in which the dependent variable, child polio non-vaccination status, is expected to follow a normal distribution with the prior of the coefficients normally distributed as non-informative priors. It is assumed that the regression parameters of concern will follow a normal distribution with mean = 0 and precision = 0.001. Three chains of parameters were simulated for 50,000 iterations each, and a total posterior sample of 90,000 is kept for summarization and convergence checks after discarding the first 20,000 iterations as burn-in. Evaluation of Model Convergence of MCMC was checked by a time-series plot, Gelman–Rubin statistic, autocorrelation plot, and kernel density plot, with all plots showing that the convergence of the model was achieved. The Bayesian approach usually reports either the mean or median of the posterior samples for each parameter of concern as a point estimate.

## 3. Results 

### 3.1. Descriptive and Test of Association Results

Out of 2016 total children aged between 12 and 23 months included in the study, 881 of them (about 43.7%) were not fully vaccinated against polio at the time of the survey (Table 1). From the predictors included in the model shown in Table 1, the sex of the child, maternal age, maternal education, family wealth index, occupation, family size, exposure to mass media, presence of health facility nearby, means of transport, place of residence, counseling on vaccination, and place of delivery were statistically significantly associated with incomplete vaccination against polio. 

### 3.2. Binary Logistic Regression Results

The results in Table 2 shows that maternal age, mother’s educational level, household wealth index, exposure to mass media, place of residence, presence of nearby healthy facility, counseling on vaccination, and place of delivery were the significant predictors of incomplete vaccination against polio among child aged between 12 and 23 months.

Children of mothers aged 25–34 and 35–49 were about 18% and 23% (OR = 0.82 with 95% CI = 0.74, 0.93) and OR = 0.77, with 95% CI (0.62, 0.94) less likely not to be fully vaccinated against polio compared to those of mothers in the age group 15–24. The result also indicates children of mothers who had primary and secondary and above education were about 27% (OR = 0.73 with 95% CI = 0.55, 0.93) and 37% (OR = 0.63 with 95% CI = 0.48, 0.79) less likely not to be fully vaccinated against polio compared those of mothers with no formal education. Concerning the household wealth index, children from medium and rich families were 47% (OR = 0.53 with 95% CI = 0.278, 0.873) and 58% (OR = 0.42 with 95% CI = 0.189, 0.670) less likely not to be fully vaccinated against polio compared to those from poor families. Similarly, children of mothers who are exposed to mass media once a week and twice and more a week were 17.9% (OR = 0.821 with 95% CI = 0.729, 0.915) and 32.6% (OR = 0.684 with 95% CI = 0.534, 0.836) less likely not to be fully vaccinated against polio compared to children of mothers who had no media exposure at all.

Incomplete polio vaccination among children aged 12–23 months from rural areas decreased by 24.7% (OR = 0.753 with 95% CI = 0.585, 0.925) compared with those from urban areas. The results also show that children from households that were not near a health facility were 93% (OR = 1.93 with 95% CI = 1.563, 2.315) more likely to have incomplete polio vaccination compared to children from households that were near a health facility. Similarly, children whose mothers were not counseled on child vaccination were more likely not to be fully vaccinated against polio compared to those whose mothers were counseled on vaccination (OR = 2.35 with 95% CI = 2.14, 2.59). We also found that children who were delivered at home were three times more likely not to be fully vaccinated against polio compared to those who were delivered at a health facility (OR = 3.14 with 95% CI = 2.95, 3.45) (Table 2). 

### 3.3. Bayesian Logistic Regression Results 

Approximately 2.5% and 97.5% percentiles of the posterior samples for each parameter provide a 95% posterior reliable interval (interval within which the parameter lies with probability of 0.95). The results of Table 3 indicate that maternal age, mother educational level, household wealth index, exposure to mass media, place of residence, presence of nearby healthy facility, counseling on vaccination, and place of delivery significantly contributed to the prediction of incomplete polio vaccination status among children aged between 12 and 23 months (since the credible intervals of these variables do not contain zero).

In Bayesian analysis, to have accurate posterior estimates, the simulation should be run until the Monte Carlo (MC) error for each parameter of interest is less than about 5% of the sample standard deviation. In Table 3, the MC errors for all the parameters were less than 5% of its posterior standard error. This approach suggests that the convergence and accuracy of the posterior estimates are achieved. Both the classical parameter estimation approach and Bayesian parameter estimation approach fit the data well and give almost consistent results, but most of the parameters in the Bayesian analysis had smaller errors than the corresponding classical binary logistic model, and the Bayesian analysis provides additional solutions as the posterior distribution of the parameters.

## 4. Discussion

This study examines the predictors of incomplete vaccination against polio among children between 12 and 23 months in Ethiopia. The results showed that maternal age, mother’s educational level, household wealth index, exposure to mass media, place of residence, presence of nearby healthy facility, counseling on vaccination, and place of delivery were associated with incomplete polio vaccination among children aged 12–23 months in Ethiopia.

Concerning maternal age, it was found that the odds of incomplete vaccination against polio decreases with increasing age. Hence, children born to younger women were more likely to have incomplete vaccination against polio compared to those born to older women. This finding is consistent with the findings of studies that have been carried out in Ethiopia [26,27] and other low-and middle-income countries [28,29,30,31], which found that young maternal age is a risk factor for incomplete childhood immunization. The possible reason for the finding could be the better understanding of the child’s health and the importance of vaccination among older women compared to younger women [30,31]. Younger women may also face challenges accessing health care to immunize their children including stigma, cost, and distance to health facilities [32,33,34]. This finding calls for the need to address barriers to maternal healthcare services utilization among young mothers. There is also the need to educate young mothers on the importance of vaccination against polio to a child’s survival.

Children of mothers with a higher educational level and wealth index were less likely to be incompletely immunized against polio compared with those with no formal education. High socio-economic status (wealth and educational level) has been found to be protective against incomplete childhood vaccination in several studies in low- and middle-income countries, including Ethiopia [26,27,28,29,30,31]. In explaining the relationship between socio-economic status and childhood vaccination, several authors have explained that higher socio-economic status enhanced health-care seeking for child health, including immunization [35,36,37]. Specifically, women with a higher level of education were considered as more knowledgeable of the risks associated with incomplete childhood vaccination and the importance of complete childhood vaccination [35,36,37]. The knowledge obtained by women with higher levels of education is available to them not only through schooling but also through exposure to media, where information about the risks associated with incomplete vaccination and the importance of complete childhood vaccination is available [35,36,37]. Those with a higher wealth quintile were found as having the economic empowerment to overcome the financial cost of accessing health care for their children and thus are able to access health care for their children [29,31,38]. Unless they live in areas where childhood vaccinations are offered to children in their homes, the place where a mother lives also plays a major role in the incomplete vaccination of children. However, in this study, children of mothers who lived in rural areas were less likely to be incompletely immunized compared to those who lived in urban areas. This finding contradicts previous studies that explained that access to nearby health facilities in urban areas reduced incomplete vaccination against polio [29,30,39,40]. The findings underscore the importance of addressing socio-economic inequalities in access to maternal and child health-care services. Special attention should be given to mothers of high socio-economic disadvantages by reducing the cost of child health care and educating those with no formal education on the importance of childhood vaccination against polio. Health-care workers in Ethiopia may consider providing community-based vaccination services that will target mothers who live in rural areas and far away from health facilities.

Children who were delivered at home were more likely to be incompletely vaccinated against polio compared to those who were delivered at the health facility. Previous studies have shown that maternal health-care utilization plays a key role in determining the complete vaccination status of children [26,30,35]. In relation to the observed association between place of delivery and incomplete vaccination against polio among children in Ethiopia, women who deliver at health facilities might be exposed to information about their health and that of their children [41]. Such information may include vaccination counseling [42], which was also found to decrease the likelihood of incomplete polio vaccination in the current study. Thus, women who deliver in health facilities are likely to receive adequate training from health professionals on the importance of childhood vaccinations, and this builds their confidence in using preventive health services [30]. This finding calls for the need to educate pregnant women during antenatal care attendance on the importance of health facility delivery for maternal and child health and its impact on childhood vaccination against polio specifically. Notwithstanding, as long as some women continue to deliver at home, special attention should be given to reach these children and provide them with the necessary vaccinations.

### Strengths and Limitations

The robust analytical approaches employed in the analysis of data for the current study constitutes its key strength. We also followed a systematic methodological process in arriving at our findings and conclusion, making our findings and conclusions reliable. The use of nationally representative data also makes our findings generalizable. Nonetheless, our results should be interpreted with caution, since the cross-sectional nature of the data makes it impossible for us to establish causality. Some of the mothers also gave verbal responses about the vaccination status of their children, and this can be influenced by recall bias.

## 5. Conclusions

The purpose of the paper was to examine the factors associated with incomplete vaccination against polio among children aged between 12 and 23 months in Ethiopia in order to make recommendations that will help eradicate poliovirus from the country. The prevalence of polio incomplete vaccination against polio was 43.7%. Maternal age, mother’s educational level, household wealth index, exposure to mass media, place of residence, presence of nearby healthy facility, counseling on vaccination, and place of delivery were the significant predictors of incomplete vaccination against polio among child aged between 12 and 23. Thus, the stakeholders must give consideration to all significant predictors revealed in the results of this study. The study also shows that children born to mothers with no education have a high probability of not being fully vaccinated. Therefore, the government should pay attention to maternal education to increase mothers’ educational levels in all regions and give training and counseling in all urban and rural parts of the country on child vaccination to overcome the problems of child incomplete vaccination against polio or polio vaccination dropout.

## Figures and Tables

**Table 1:** Descriptive and test of association results on polio non-vaccination status with predictor variables.

Variables	Categories	Polio Vaccination Status				Chi-Square
		Not Fully Vaccinated		Fully Vaccinated		
		Count	%	Count	%	
Sex of child	Male	409	43.7%	525	56.3%	53.98 *
	Female	473	43.7%	609	56.3%	
Maternal age	15–24	300	48.4%	320	51.6%	69.35 **
	25–34	352	43.8%	452	56.2%	
	35–49	230	38.9%	362	61.1%	
Wealth index	Poor	704	58%	200	42%	84.65 **
	Middle	249	39.1%	388	60.9%	
	Rich	177	34%	298	66%	
Occupation	House wife	619	63.8%	352	36.2%	43.24 *
	Employee	56	27%	148	73%	
	Daily labor worker	175	48%	189	52%	
	Merchant	172	36%	305	64%	
Mother educational level	No education	724	62.5%	436	37.5%	41.71 *
	primary	251	39.6%	381	60.4%	
	Secondary and above	66	29%	158	71%	
Exposure to mass media	Not at all	216	58%	158	42%	55.25 *
	Once a week	349	41%	501	59%	
	Twice and more a week	253	32.1%	536	67.9%	
Family size	1–3	370	49.8%	372	50.2%	47.18 *
	4–6	371	43.2%	485	56.8%	
	Above 7	167	38.1%	251	61.9%	
Place of residence	Urban	683	53.8%	581	46.2%	77.32 **
	Rural	271	33.6%	481	66.4%	
Presence of nearby health facility	Yes	381	35.9%	678	64.1%	65.84 **
	No	492	51.5%	465	48.5%	
Type of nearby health facility	Health center	319	42.3%	435	57.7%	2.56
	Health post	341	39.2%	528	60.8%	
	Private clinic	195	49.6%	198	50.4%	
Means of transport	On walk	497	48.3	532	51.7	36.17 *
	Transportations	387	39.1	600	60.9	
Counseling on vaccination	Yes	311	22.2%	1082	77.8%	88.59 **
	No	406	65.2%	217	34.8%	
Place of delivery	Health facility	409	31.4%	889	68.6%	40.64 *
	Home	402	56%	316	44%	

* Chi-square was significant at (*p* < 0.05) and ** Chi-square was significant at (*p* < 0.01).

**Table 2:** Results of binary logistic regression analysis.

Variables	Categories	B	S.E	Wald	Sig	Exp (B)	95%CI for Exp(B)	
							Lower	Upper
Sex of child	Female (ref) Male	−0.658	0.097	−1.13	0.071			
						0.85	0.318	1.465
Maternal age	15–24 (ref)							
	25–34	−1.172	0.112	−15.56	0.002	0.82	0.74	0.93
	35–49	−1.195	0.164	−17.42	0.001	0.77	0.62	0.94
Mother educational level	No education (ref)							
	Primary school	−2.181	0.157	−6.512	0.001	0.73	0.55	0.93
	Secondary and above	−2.528	0.127	−7.094	0.007	0.63	0.48	0.79
	Poor (ref)							
Wealth index	Middle	−0.896	0.842	−4.13	0.023	0.53	0.278	0.783
	Rich	−0.849	0.72	−5.03	0.018	0.42	0.189	0.670
Occupation	House wife (ref)							
	Employed	−0.049	1.1088	−0.92	0.146	0.650	0.24	1.060
	Labor worker	0.486	1.100	1.05	0.138	1.226	0.88	2.046
	Merchant	0.665	1.213	0.830	0.094	1.145	0.780	2.175
Family size	1–3 (ref)							
	4–6	1.001	0.846	1.39	0.083	1.767	0.570	1.131
	Above 6	0.232	0.573	1.104	0.065	2.861	0.411	1.311
Expose to mass media	Not at all (ref)							
	Once a week	−0.172	0.089	−3.16	0.019	0.821	0.729	0.915
	twice and more a week	−0.293	0.094	−4.41	0.002	0.684	0.534	0.836
Place of residence	Urban (ref)							
	Rural	−0.409	0.122	−11.239	0.001	0.753	0.585	0.925
Presence of nearby health facility	Yes (ref)							
	No	1.62	0.856	6.15	0.02	1.93	1.563	2.315
Means of transport	Transportations (ref)							
	On walk	2.654	0.765	0.85	0.985	0.856	0.15	1.861
Counseling on vaccination	Yes (ref)							
	No	1.49	0.196	3.65	0.035	2.35	2.14	2.59
Place of delivery	Health institution (ref)							
	Home	0.870	0.791	4.21	0.029	3.14	2.95	3.45

**Table 3:** Results of Bayesian binary logistic regression analysis.

Variables	Categories	Mode	Estimate	Sd	MC Error	2.5%	Median	97.5%
Intercept		Alpha	0.1485	0.0728	0.007515	0.2970	0.5485	0.8535
Sex of child	Female (ref)							
	Male	beta [1]	−0.655	0.0920	$6.734\times 10^{-5}$	−1.025	−0.659	0.175
Maternal age	15–24 (ref)							
	25–34	beta [2]	−1.14	0.112	$2.794\times 10^{-5}$	−1.82	−1.138	−0.456
	35–49	beta [3]	−1.186	0.158	$1.581\times 10^{-4}$	−1.43	−1.185	−0.940
Mother educational level	No education (ref)							
	Primary	beta [4]	−2.181	0.0131	0.0001522	−2.97	−2.20	−1.43
	Secondary and above	beta [5]	−2.521	0.125	0.000532	−2.82	−2.53	−2.240
	Poor (ref)							
Wealth index	medium	beta [6]	−0.894	0.0839	$1.156\times 10^{-5}$	−0.983	−0.885	−0.787
	Rich	beta [7]	−0.846	0.0721	$1.458\times 10^{-4}$	−0.989	−0.848	−0.707
Occupation	House wife (ref)							
	Employed	beta [8]	−0.051	0.108	0.0002478	−0.276	−0.051	0.174
	Labor worker	beta [9]	0.487	0.105	$4.195\times 10^{-5}$	−0.258	0.456	0.979
	Merchant	beta [10]	0.668	0.0211	0.000243	−0.154	0.675	1.358
Family size	1–3 (ref)							
	4–6	beta [11]	1.001	0.0845	$6.395\times 10^{-5}$	−0.253	1.03	1.875
	Above 6	beta [12]	0.231	0.574	$3.164\times 10^{-4}$	−0.125	0.242	0.648
Expose to mass media	Not at all (ref)							
	Once a week	beta [13]	−0.74	0.092	$5.809\times 10^{-6}$	−0.98	−0.75	−0.520
	Twice and more a week	beta [14]	−0.290	0.091	$2.985\times 10^{-4}$	−0.428	−0.291	−0.152
Place of residence	Urban (ref)							
	Rural	beta [15]	−0.407	0.119	$5.239\times 10^{-5}$	−0.635	−0.411	−0.187
Presence of nearby health facility	Yes (ref)							
	No	beta [16]	1.67	0.449	0.00056	1.362	1.618	1.874
Means of transport	On walk (ref)							
	Transportations	beta [17]	2.653	0.082	0.0007893	−0.162	2.684	5.306
Counseling on vaccination	Yes (ref)							
	No	beta [18]	1.46	0.0470	$5.214\times 10^{-5}$	1.025	1.471	1.917
Place of delivery	Health institution (ref)							
	Home	beta [19]	0.864	0.0650	$4.665\times 10^{-6}$	0.435	0.859	1.283

## Data Availability

The dataset is available on the following website: https://dhsprogram.com/data/dataset/Ethiopia_Standard-DHS_2016.cfm?flag=0 (accessed on 6 October 2021).
